# New insights on the evolutionary relationships between the major lineages of Amoebozoa

**DOI:** 10.1038/s41598-022-15372-7

**Published:** 2022-07-01

**Authors:** Yonas I. Tekle, Fang Wang, Fiona C. Wood, O. Roger Anderson, Alexey Smirnov

**Affiliations:** 1 Department of Biology, Spelman College, 350 Spelman Lane Southwest, Atlanta, GA 30314 USA; 2 Department of Biology and Paleo Environment, Lamont‐Doherty Earth Observatory of Columbia University, Palisades, NY USA; 3 Department of Invertebrate Zoology, Faculty of Biology, St. Petersburg State University, Saint Petersburg, Russia

**Keywords:** Phylogenetics, Taxonomy

## Abstract

The supergroup Amoebozoa unites a wide diversity of amoeboid organisms and encompasses enigmatic lineages that have been recalcitrant to modern phylogenetics. Deep divergences, taxonomic placement of some key taxa and character evolution in the group largely remain poorly elucidated or controversial. We surveyed available Amoebozoa genomes and transcriptomes to mine conserved putative single copy genes, which were used to enrich gene sampling and generate the largest supermatrix in the group to date; encompassing 824 genes, including gene sequences not previously analyzed. We recovered a well-resolved and supported tree of Amoebozoa, revealing novel deep level relationships and resolving placement of enigmatic lineages congruent with morphological data. In our analysis the deepest branching group is Tubulinea. A recent proposed major clade Tevosa, uniting Evosea and Tubulinea, is not supported. Based on the new phylogenetic tree, paleoecological and paleontological data as well as data on the biology of presently living amoebozoans, we hypothesize that the evolution of Amoebozoa probably was driven by adaptive responses to a changing environment, where successful survival and predation resulted from a capacity to disrupt and graze on microbial mats-a dominant ecosystem of the mid-Proterozoic period of the Earth history.

## Introduction

The supergroup Amoebozoa^1^ comprises a variety of amoeboid lineages; namely, naked lobose amoebae (which are “archetypal” amoebae), testate lobose amoebae, mycetozoans, anaerobic archamoebians and a heterogeneous assemblage of flattened amoeboid, branching reticulate or flagellated organisms; presently known as Variosea. Amoebozoa holds a key evolutionary position, being the closest known relative of Obazoa that, among other organisms, includes humans^2,3^. Resolving the phylogenetic tree of this lineage is critical for answering important questions pertaining to the evolutionary origin of Amoebozoa, as well as for further clarification of the root of the eukaryotic tree^3–8^.

Our understanding of the evolution and taxonomy of amoeboid protists was originally conceived from cytological, morphological and life cycle evidence^9,10^. Early studies based on small subunit rDNA (18S) gene indicated the polyphyly of naked amoebae (gymnamoebae) and formed the basis of our understanding of the supergroup Amoebozoa^1,11,12^. The assemblage of Amoebozoa grew in membership, albeit with little improved resolution; or sometimes with conflicting hypotheses pertaining to within-group relationships (e.g.,^13–19^). This led to subsequent revisions and reevaluation in attempts to combine morphological and molecular characters and find synapomorphic characters of major clades^20–23^. While this achieved major progress in our overall understanding of the group, much of the deep and intermediate relationships and placement of some groups of uncertain phylogenetic affinities (so-called *incertae sedis* taxa) remained elusive. Multigene studies, varying in breadth and depth of gene and taxon sampling, managed to overcome many of the challenges of single-gene reconstructions; and they resolved some of the long-standing evolutionary questions in the group^4,24–27^. A recent phylogenomic study by Kang et al.^4^ reported a deep level phylogeny of Amoebozoa based on large taxon sampling. However, the placements of some *incertae sedis* lineages were not entirely resolved. For some groups, other phylogenomic studies reported conflicting relationships^25,26,28^.

The conflict in existing phylogenomic studies can be attributed partially to limitations of taxon and gene sampling as well as the methodology. Kang et al.^4^ used large taxon sampling, but included only a small fraction of data (325 genes), from the vast amount of transcriptomic and genomic data available, based on commonly used genetic markers in eukaryotes. There are data suggesting that taxon sampling alone is not sufficient to resolve deep divergences in ancient lineages that might have undergone rapid radiations^29^. The age of Amoebozoa is estimated to be over a billion years, and the probable origin of the group is dated back to the mid-Proterozoic period^30,31^. Therefore, in order to infer deep evolutionary divergences not only increased taxon sampling, but also more representative genetic sampling along with the application of appropriate models and methods, are essential.

In this study, we sampled putative single copy gene markers from genome-wide assays, increased taxon sampling to include genes not previously used in Amoebozoan phylogenomics, and produced the largest amoebozoan supermatrix to date. This large dataset enabled us to recover a well-resolved and supported tree of the Amoebozoa. In addition, we uncover a well-corroborated novel deep-level relationship and resolved the placement of some *incertae sedis* lineages.

## Results

### The tree of amoebozoa

**Figure 1:**
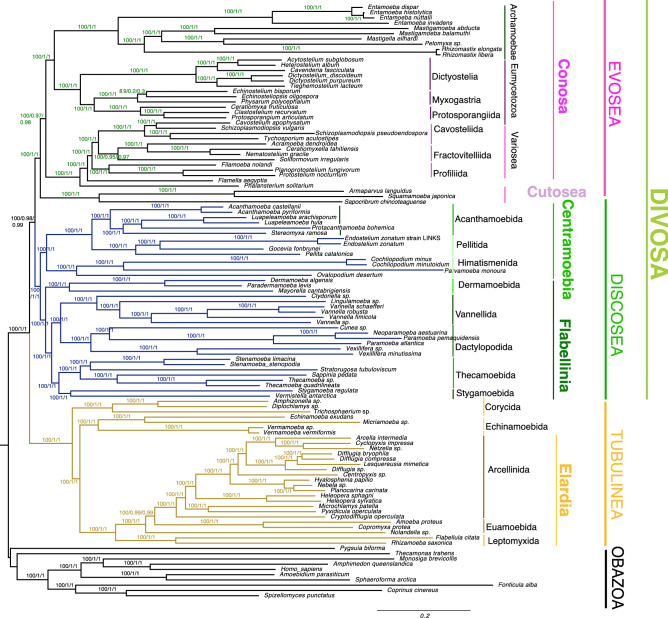
Genome wide phylogeny of the Amoebozoa inferred using Maximum likelihood (ML) in IQ-TREE with LG + G4 + C60 + F model of evolution. The data matrix used to infer this tree consisted of 113,910 amino acid sites from the full dataset, derived from 824 genes and 113 taxa including 10 outgroup taxa. Clade supports at nodes are ML IQ-TREE 1000 ultrafast bootstrap values, obtained using the same model, Internode certainty inferred using QuartetScores and RAxML, respectively. All branches are drawn to scale except branches leading to Archamoebae, and *Sapocribrum chincoteaguense,* and *Parvamoeba monoura*, that were reduced to one-third and half, respectively.

We compiled the largest supermatrix consisting of 824 genes, including over 450 novel genes not previously used in the group, based on genome wide gene assay to reconstruct the phylogeny of the Amoebozoa. We recovered a monophyletic tree of Amoebozoa that is well resolved and supported in every one of our analyses (Figs. 1, S1–S4). Our datasets, with and without fast-evolving sites removed (analyzed using the complex amino acid model, LG + G4 + C60 + F, in IQ-TREE), recovered all well-established major subclades of Amoebozoa including Discosea, Archamoebae, Cutosea, Eumycetozoa, Variosea and Tubulinea with full support (Figs. 1, S1). The two well-known long-branch lineages, Archamoebae and Cutosea, were placed in their respective established phylogenetic positions, without removal of fast evolving sites in our full dataset (Fig. 1). Removal of fast evolving, rate categories, in IQ-TREE neither affected the topology nor improved support values (Fig. S1). In the RAxML analysis, the accurate placement of Archamoebae and Cutosea, required removal of six fast evolving rate categories (38%) from the full dataset (Fig. S2); but resulted in the same final tree configuration. The RAxML tree had generally lower ML bootstrap supported branches but was congruent with the topology of the trees inferred using IQ-TREE (Figs. 1, S1, S2). A similar reduced dataset was analyzed using Bayesian inference, which yielded similar topology despite lack of convergence in our PhyloBayes analysis (data not shown). Kang et al.^4^ also reported similar topologies among their ML and PhyloBayes trees despite limited number of chains used and lack of convergence in some of their PhyloBayes analyses. Due to the high computational demand, Bayesian inference was not feasible with our large dataset. The consistency of tree topologies across methods and algorithms used, as well as the placement of long-branch taxa (Archamoebae and Cutosea) without removal of fast evolving sites in IQ-TREE (likely due to complex model used), demonstrates the robustness of our result.

In our phylogenomic tree, all major clades are congruent with previous published topologies^4,24–26^. Moreover, our phylogenomic tree has well-corroborated relationships; and the recovery and placement of enigmatic taxa are more stable (Figs. 1, S1, S2). Our results yielded improved support for the Flabellinia and Thecamoebida clades compared to a previous comparable phylogenomic study^4^. We have recovered for the first time a fully supported monophyletic clade encompassing two *incertae sedis* taxa, *Vermistella* and *Stygamoeba*. Both these lineages were placed in the order Stygamoebida based on morphological evidence^22^. The monophyly and placement of this order in the tree of Amoebozoa has not been resolved in previous multigene analyses (e.g.,^4^). In our tree Stygamoebida clade forms a sister group relationship with Thecamoebida with full support (Fig. 1). We also find some discrepancies between our tree (Fig. 1) and that of Lahr et al.^5^ in the branching order of the Tubulinea clade, albeit with similar taxon sampling for this clade. Our analysis (Fig. 1) shows clade Corycida as the most basal Tubulinea lineage similar to that of the Kang et al.^4^ phylogeny, while in Lahr et al.^5^ Echinamoebidae is shown as the most basal tubulind lineage. *Nolandella* sp., a member of Euamoebida, did not group with *Amoeba proteus* and *Copromyxa protea* in our analysis, but formed an independent lineage (Fig. 1).

### A novel deep split of the amoebozoa

Our analysis for the first time revealed a novel, well-supported deep spilt of Amoebozoa; not reported in previous phylogenomic studies. Amoebozoa is split into two fully supported major subclades: Tubulinea and a second one comprised of the remaining major subclades including Evosea (Eumycetozoa, Variosea, Archamoebae, and Cutosea) and Discosea (Figs. 1, S1, S2). This branching is different from a finding of Kang et al.^4^, a recent phylogenomic study that reported a spilt between Discosea and Tevosa (Evosea + Tubulinea)^4^. Tevosa is not supported in our analyses, including analyses with removal of fast evolving sites. On the other hand, the deep split (Evosea + Discosea *vs.* Tubulinea) observed in our phylogenomic tree is supported in all analyses of our data sets. The deep spilt receives almost full support in our internode certainty (IC) analyses as implemented in QuartetScores (1.00) and RAxML (0.979) (Figs. 1, S3, S4). AU test of our topology, comparing alternative topologies with Tevosa and a traditional deep relationship uniting Discosea and Tubulinea (Lobosa), showed that the newly recovered deep spilt has the highest p-value (p-AU = 0.947). The hypothesized group Lobosa was rejected (p-AU = 0.000278), while Tevosa cannot be rejected with p-value just above threshold (p-AU = 0.0564). For convenience, we suggest a new name for the deep spilt (Discosea + Evosea) clade; i.e., Divosa, a term derived from a combination of the name of the two clades.

## Discussion

### Targeted genome-wide data enrichment for phylogenomics of amoebozoa

Despite the large number of RNA-Seq data generated in recent studies^4,24–26^, only a small fraction of this data has been utilized in phylogenomic analyses. To increase it, we compiled a total of 1559 markers using genome-derived protein coding genes from 113 amoebozoan genomes and transcriptomes. Usage of putative single copy markers, primarily derived from Amoebozoa genomes, has enabled us to introduce highly conserved markers with phylogenetic signal corroborating morphology-based and phylogenomic-based amoebozoan hypotheses^4,24^. While single-copy genes identified in some genomes might not always apply to others, a previous phylogenomic study with seed plants, based on single copy markers resulted in more resolved phylogeny both at shallow and deep nodes^32^. In this study, we followed a stringent approach aided by automated and manual curation of markers, selected from the above-mentioned dataset to build the largest supermatrix (824 genes) in the Amoebozoa. With this approach, we substantially increased the total number of genes used in Amoebozoa phylogenomics. Our analysis yielded consistent and well-corroborated topologies, despite whether we included or excluded fast evolving sites (Figs. 1, S2). The robustness of our phylogeny is also corroborated with the high support values from internode certainty analysis (Figs. 1, S3, S4). One of the evident results of this approach is the first time phylogenomic recovery of the monophyly of the taxon Stygamoebida, earlier supported only at the morphological level^22,23^ and a recovery of a novel deep split divergence of Amoebozoa.

### Unraveling deep divergence of amoebozoa

A recent phylogenomic study by Kang et al.^4^, though based on a slightly smaller taxon sampling, proposed a split of the Amoebozoa supergroup into two major subclades: Tevosa (Evosea + Tubulinea) and Discosea. By contrast, in our study Evosea robustly groups as sister clade to Discosea (Figs. 1, S1, S2). Both phylogenetic hypotheses, ‘Tevosa’ and Divosa, receive high statistical support in their and our study, respectively (see Fig. 1). In^4^ phylogenomic analyses, it is common to see that short subtending deep nodes receive high statistical support^33^. Amoebozoan deep nodes are characterized by very short branch lengths, an indication of limited supporting characters, or possible ancient rapid diversification. Strong statistical support at these levels of nodes does not necessarily mean that the inferred relationships are correct. Statistical indices such as bootstrap values and Bayesian posterior probabilities only assess sampling effects, and give an indication of tree reliability that is dependent on the data and the method^34^. This can partially explain why these short-branch, deep nodes in Amoebozoa phylogenomic studies tend to collapse, or vary, depending on the method of analysis or the composition of the gene/taxon sampling^4,24–26^. Certainly, caution still must be taken when interpreting ancient divergences, because results can be muddied by noise (e.g., gene history^35^ or lack of signal due to rapid radiation^29^). However, the support of the split recovered in the present study is high and originates from different lines of evidence. While we have used relatively decent taxon sampling in our study, the resolution of deep nodes will improve with inclusion of lineages that are underrepresented (e.g., Cutosea) and discovery of new deeply branching lineages within the Amoebozoa.

We also note that in many lineages trophozoites of Discosea and Variosea are more similar to each other rather than to Tubulinea. Certainly, the morphology of presently living amoeboid organisms is derived and adaptive, but generally it is possible to say that members of Divosa lineage share more morphological similarity between each other rather than with the Tubulinea lineage. For example, amoebae of the genus *Flamella,* belonging to the class Variosea, by their morphology may be easily confused with some discosean amoebae (e.g., see Michel and Smirnov^36^); the same is true for individual trophozoites of many mycetozoan species, showing flattened body shape and pointed subpseudopodia^37,38^. Cells of amoebae belonging to the genus *Squamamoeba* (the taxon of Cutosea), sometimes resemble *Korotnevella* (Discosea) in their overall morphology; hence, being differently organized at the cytological level^39^. At the same time, none of discosean or variosean lineages show the morphology resembling that of, e.g., Amoebida, or alteration of the locomotive morphology from flattened to tubular, which is a general characteristic of Tubulinea^20,22^. The return to the earlier-derived tubular body shape, subcylindrical in cross-section, occurs among amoeboid representatives of Archamoebea; however, this might be mostly an adaptation related with their specific lifestyle (parasites or pelobionts). In addition the pattern of pseudopod formation (e.g., the tendency to show eruption of the hyaline cytoplasm in the frontal area of the cell) makes them to be significantly different from that in Tubulinea (see^40^). However, three species belonging to shallow nodes of the Discosea clade show tubular morphology^41–43^. Kudryavtsev et al.^43^ reasonably suggested that this may indicate that tubular body form might be a plesiomorphic character for the Amoebozoa.

### Mid-Proterozoic environment–the driving force for the origin of amoebozoa

The flagellum (cilium) is a highly conserved complex structure that is believed to have originated only once, and be ancestral to all eukaryotes^2,44,45^. Amoebozoa are remarkable in that the two basal phylogenetic lineages, Tubulinea and Discosea, have entirely lost cilia, kinetosomes (basal bodies), and associated root structures; while a derived major clade, Evosea, contains a handful of ciliated lineages in a few branches intermingled among amoeboid lineages^21,22^. The loss of cilia and associated structures in the majority members of Amoebozoa is one of the biggest mysteries pertaining to their origin and evolution.

In ciliated members of Amoebozoa, the ciliary apparatus is characterized by a specific arrangement of root structures, which includes an incomplete (Variosea and Mycetozoa) or complete (Archamoebea) cone of microtubules extending from the kinetosome to the nucleus^46^. In early interpretations, this conical arrangement of microtubules was considered to be homologous to the ciliary root system of Opisthokonta; which, together with other morphological and molecular evidence, gave rise to the “Unikonta” hypothesis^2,47,48^. In this model, the hypothetical ancestor of Amoebozoa was considered to be an organism with a single emergent cilium, resembling *Phalansterium* or *Mastigamoeba* in cellular organization^49,50^. This lineage, combining Amoebozoa and Opisthokonta, has been proposed as an alternative to that of the bikonts, with two emerging cilia; which included the rest of the eukaryotic groups. Cavalier-Smith^2^ argued that among unikonts, paired kinetosomes (when present) resulted from convergent evolution rather than common ancestry with bikonts. Molecular and morphological analyses provided certain indications that the microtubular structures in Amoebozoa, and Opisthokonta may not be homologues^46,51^. However, further development of molecular phylogeny provided evidence for the basal position of bikont organisms in the tree of eukaryotes^3,52,53^. Thereafter, the general consensus nowadays is that the hypothetical common ancestor of Amoebozoa was a bikont organism^46,54,55^. Several authors (e.g.,^3,46,52,53^) hypothesised that the presumable common ancestor was a ventrally-grooved biciliate gliding flagellate, capable of producing filose ventral pseudopodia and possessing a relatively complex organization of the cell. That is, a cell possessing two cilia with kinetosomes and root structures, ventral groove supported with microtubules and dorsal pellicle-the so called “sulcozoan ancestor”. Its name originates from Sulcozoa-a phylum of protists established by Cavalier-Smith^46^ that combines a heterogenous assemblage of early evolving eukaryotic lineages. Cavalier-Smith suggested that “opisthokonts and Amoebozoa evolved from sulcozoan ancestors by two independent losses of the pellicular dense layers and of the ventral groove, which in both cases would allow pseudopods to develop anywhere on the cell surface” (op. cit.).

**Figure 2:**
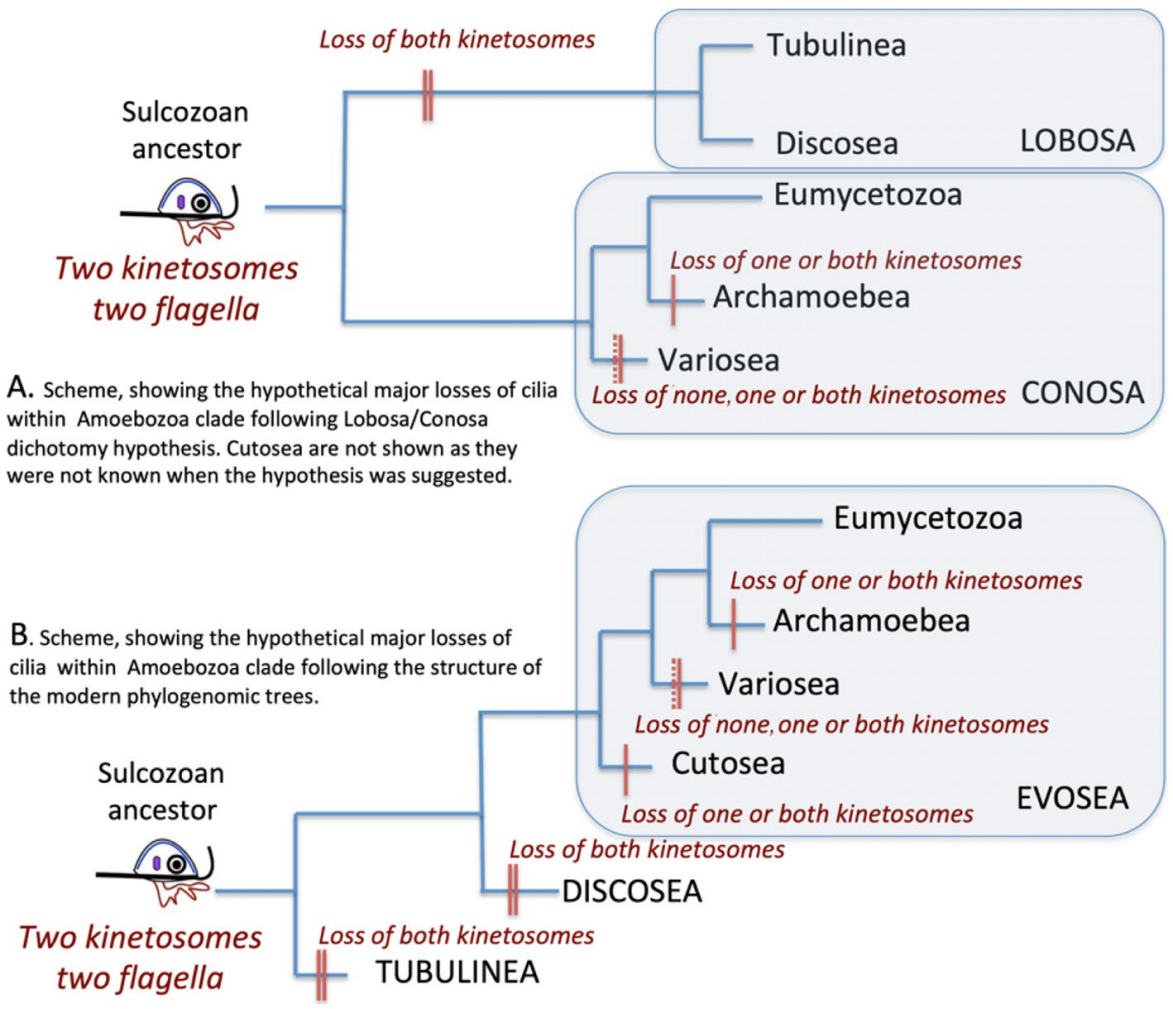
A scheme illustrating the loss of kinetosomes and cilia under the different configurations of the phylogenetic tree (**A** and **B**). Vertical hash marks on branches show loss of kinetosomes (the number lost as designated by labels on the diagram) depending on the lineage.

The origin and further evolution of Amoebozoa in this hypothesis presumes the loss of both cilia and kinetosomes in Lobosa (Tubulinea and Discosea) and of the posterior cilium and one kinetosome in most of the ancestors of Conosa—Archamoebae, Variosea and Eumycetozoa; Cutosea were not known at that time (e.g.,^3,52,53^). This evolutionary events was rather logical and is illustrated in Fig. 2A. However, the Lobosa/Conosa dichotomy was doubted based on some 18S gene phylogenies^27^; and it subsequently failed to garner support in wide-scale phylogenomic studies^4,24,25^, as well as in the present study. This makes the model of multiple losses more complicated; because under the new tree configuration, we have to suggest subsequent partial or complete loss of cilia and related structures in all but one branch of Amoebozoa. This sequence of losses is illustrated in Fig. 2B. It remains unclear why the hypothesized ancestor of Amoebozoa, being initially a quite complex biciliated organism, underwent such a massive loss (or substantial simplification) of cilia-related structures in almost all evolutionary lineages of Amoebozoa, and what was the driving force for such a reduction.

Several studies based on molecular dating analysis correspondingly placed the origin of Amoebozoa to the Mesoproterozoic period, which means 1250–1624 mya^31,56^. It means that the early evolution of Amoebozoa took place at the period when the biosphere was dominated with microbial biofilms-sheets of bacteria, embedded in extracellular polymeric substances, covering almost every possible substrate^57^. Being initially rather simple, biofilms further evolved to become complex microbial mats, comprising different prokaryotic and microeukaryotic organisms, showing concerted activities and intimate interactions between various microbial metabolisms^58^. The oldest mats are dated to approximately 3.5 billion years ago, and the noonday of mats covers the mid-Proterozoic period^59,60^, which roughly corresponds to the estimate of the potential age of Amoebozoa.

Formation of a microbial biofilm, among other structural and biogeochemical features, can be explained as an adaptation that increases survival of bacteria to avoid predation^61,62^. The probable size of the bacterivorous biflagellate ancestor of Amoebozoa was relatively small, likely no larger than that of the existing representatives of the CRuMs clade (e.g., *Mantamonas*) or ‘Excavates’ (metamonads or *Malawimonas*), which is within the general size range of 2–20 µm. These organisms would be capable of preying on solitary bacteria, but consumption of larger microorganisms embedded in an intact microbial mat probably was beyond their capacity, as well as this is beyond the capacity of the modern flagellates of comparable size^63,64^. Feeding on bacteria, major constituents of the microbial mats (the dominant food source in the mid-Proterozoic environment), was facilitated by adaptations to invade and prey on the mat microbial communities, including increment in the body size and acquisition of special adaptations allowing them to ingest filamentous food. However, the latter was again related to the body size, because the filament, even compacted in some way, must be ingested-i.e., appear inside the cell.

Due to Reynolds number limitation^65,66^, the increment in the body size makes ciliary motility less adaptive due to loss of efficiency. Thus, from an adaptive aspect, an amoeboid lifestyle might be a way to increase the body size while retaining a motility function, no longer dependent on cilia. An amoeboid organization also could gain the adaptive capacity to disrupt microbial mats and graze, feeding on bacteria within the mats. This adaptation would provide access to the dominant food source in the biosphere of the mid-proterozoic eon. Indeed, presently, naked amoebae are known as one of the primary grazers of bacterial biofilms^67–69^. Moreover, they not only just graze and phagocytize prey in the mats, but also disrupt them, making their content available for other organisms^70,71^. Finally, in addition to the advantage of feeding on bacterial mats^72,73^, it is also possible that an increase in body size alleviated pressure of predation by other organisms on the last Amoebozoan common ancestor (LACA), which for some time provided it an adaptive advantage and allowed rapid proliferation and differentiation of Amoebozoa in the mid-Proterozoic environment.

Hence, we hypothesise that the adaptive value of amoeboid locomotion and concomitant grazing potential on the dominant food source in the mid-proterozoic biosphere-the microbial mats-favoured the evolution of the Amoebozoa. They probably succeeded in competing for, and preying on, bacteria in the microbial mat by an increase of body size. However, at the same time, the efficiency of flagellar locomotion was highly reduced or lost; and this resulted in the multiple suspensions of the flagellar apparatus during the course of evolution, which presently is completely absent in two major current amoebozoan lineages-Tubulinea and Discosea (Fig. 2). The modern configuration of the Amoebozoan tree, which rejects the Lobosa/Conosa dichotomy and suggests a subsequent branching of lineages (with either Tubulinea or Discosea at the base), leaves open a major question. That is, was the last Amoebozoa common ancestor an amoeboflagellate, with the domination of amoeboid movement based on the microtubular cytoskeleton; or was the flagellum-related structures and microtubular locomotive system entirely suppressed? If the latter case is true, then an adaptive response that enhanced amoeboid locomotion based on an acto-myosin movement would have promoted survival and success of the protist, and lead to the current form of amoeboid locomotion as found in modern representatives of naked and testate lobose amoebae. Probably, the answer to this question may be obtained by the analysis of gene content and the level of flagellum-related gene expression in the amoebozoan genomes. However, the dataset available for quality analysis remains limited in this group of protists and requires further accumulation prior to conclusive study.

## Methods

### Transcriptome assembly and contamination examination

All transcriptome data used in this study were assembled using a bioinformatics pipeline described in Tekle and Wood^25^. As a precautionary measure for contamination, high-quality data generated from single-cell or monoclonal cultures, and without history of contamination, were prioritized in our data collection. We also checked highly conserved genes (e.g., small subunit rDNA and cytoskeletal genes) for assembled transcriptomes to check the identity of the species. Species suspected to have been contaminated (e.g., *Ripella* sp. DP13-Kostka) or with low- or poor-quality transcriptome data (see below) have been removed from the final analysis. Assembled contigs were translated into protein sequences using TransDecoder (https://github.com/TransDecoder/TransDecoder/wiki).

### Taxon and gene sampling

A total of 107 amoebozoans representing the vast diversity of the supergroup and 10 outgroup taxa from a closely related clade, Obazoa, were included in our initial analysis (Table S1). Four ingroup taxa including *Parvamoeba rugata*, *Centropyxis aculeata*, *Hyalosphenia elegans* and *Grellamoeba robusta,* were removed from the final dataset due to poor data quality. A recent phylogenomic study^5^ that focused on testate amoebae (clade Tubulinea) reported a topology of Tubulinea that differed from that of Kang et al.^4^. To explore these discrepancies further, and assess the impact of taxon sampling on the branching order of Tubulinea clade and its position within the Amoebozoa phylogeny, we added more slowly evolving taxa to Tubulinea. The final supermatrix consisted of 113 taxa including the outgroup taxa (Table S1).

A genome wide gene sampling approach using available amoebozoan genomes was employed to identify single copy markers. Previous phylogenomic studies have used conserved phylogenetic markers commonly found in a wide range of eukaryotic diversity^4,24^. In this study, we used a series of bioinformatics steps to maximize gene sampling in the Amoebozoa. We conducted a whole genome comparison of three well-annotated amoebozoan genomes, *Acanthamoeba castellanii*, *Dictyostelium discoideum* and *Entamoeba histolytica,* to extract commonly shared protein-coding genes among these genomes in OrthoVenn^74^. Inclusion of *E. histolytica* greatly reduced the number of shared genes by 40% because this amitochondriate parasitic species has a comparably much reduced genome to the free-living amoebae. For this reason, to be more representative, further comparative analysis was done using *A. castellanii* and *D. discoideum* as reference genomes to mine single-copy genes. Using this approach, we identified 1559 putative single copy genes that were used as a query to search orthologous genes from ingroup and outgroup taxa.

We used NCBI-BLAST with e-value threshold of 10^–15^ to retrieve homologous sequences from transcriptomes or genomes of our selected taxa. From this analysis, sequences with best e-value scores were retained for each taxon. The retained sequences, for each taxon and gene, were compiled and aligned using a sequence alignment tool, MAFFT, with default setting^75^. These alignments were then trimmed in TrimAl^76^ using “automated1” setting provided by the program. To inspect potential paralogs from each gene, we inferred single gene trees using IQ-TREE with the best-fit model automatically fast selected by ModelFinder^77^. Both single gene trees and their corresponding alignments were then inspected manually for paralogy and other anomalies related to alignment accuracy, sequence length and fast evolving lineages (Supplementary file 1). We applied strict gene selection criteria that included removal of anomalous grouping (e.g., lineages that grouped with outgroup or wrong (unexpected) phylogenetic position with > 90% bootstrap support) and genes that showed paralogy (duplication) signs. To mitigate the impact of long-branch attraction during phylogenetic reconstruction, we removed genes that contained three or more long-branch lineages. Long branch taxa were determined by the length of branches that are outliers relative to the remaining ingroup taxa in a phylogenetic tree. Two exceptions for this approach were the well-known long-branch lineages, Cutosea and *Entamoeba*, that were kept in all of our analyses. These two lineages were retained since all their representatives are mostly long-branches. They are also indirect indicators of noise in a data matrix since their correct placement usually requires removal of fast-evolving sites due to the effect of long-branch attraction. Following these criteria, we retained a total of 824 gene clusters in the final dataset. Orthologous group numbers were assigned for each gene cluster using ublast in USEARCH^78^ with e-value 10^–10^. We used the OrthoMCL database to generated ortholog group numbers^79^ (Table S2).

### Supermatrix construction and tree inference

The alignments from 824 genes were concatenated into an initial supermatrix containing 198,280 amino acid sites, including gene sequences not previously used in Amoebozoan phylogenomic studies, and 117 taxa using a customized R script. Taxa with over 80% gappy sites were removed, which resulted in exclusion of 4 lineages (*Parvamoeba rugata*, *Centropyxis aculeata*, *Hyalosphenia elegans*, *Grellamoeba robusta*). Constant sites, and sites with more than 50% missing data, were removed from this alignment, and the resulting supermatrix retained 113,910 amino acid sites and 113 taxa for the full dataset.

Phylogenomic analyses of the final datasets were conducted in IQ-TREE-an efficient tool to analyze large datasets by the maximum likelihood (ML) method^77^. All IQ-TREE analyses were preformed using LG + G4 + C60 + F model, with 1000 replicates for ultrafast bootstrap, which allowed full profile mixture model C60 and Gamma rate heterogeneity across sites. We also analyzed our dataset in RAxML v.8.2.X^80^ using PROTGAMMALG4X model; branch support was estimated from 1000 rapid bootstrap pseudoreplicates.

Fast-evolving sites and taxa are known to be problematic for tree inference due to saturation of substitutions and subsequent convergent evolution resulting in long-branch attraction (LBA) and other systematic errors. To test the effects of these types of errors on our phylogenomic analysis, we performed a site removal assay in which each site of the supermatrix was assigned to one of 16 categories based on its rate from IQ-TREE. This was performed using a posterior mean site frequency (PMSF) model with mixture model C60 and 16 discrete rate categories of sites. For this analysis, we used the tree from full dataset inferred above as a guide tree. The impact of fast evolving sites on resulting phylogenies was assessed by subsequent removal of fast categories of sites (up to 6 categories). In IQ-TREE our full dataset was analyzed with 3 categories removed using PMSF model with a guide tree inferred from the complex model (LG + G4 + C60 + F) mentioned above. In RAxML, 3 and 6 fast site categories were removed and analyzed using the same model as above.

### Internode certainty analysis and hypothesis testing

As an alternative to bootstrap branch support from IQ-TREE, we calculated internode certainty (IC) scores using the program QuartetScores^81^. This approach calculated IC scores from the frequencies of quartets, which can correct for the missing taxa using a set of trees. For this analysis, we used 1000 bootstrap trees generated from LG + G4 + C60 + F model in IQ-TREE with our full dataset. Alternatively, we used RAxML to estimate the degree of certainty for internodes and tree topology for bipartitions with PROTGAMMALG4X model^82^.

We used Approximately Unbiased (AU) tests^83^ to test alternate tree topologies pertaining to the deep node hypotheses Divosa (this study), Tevosa (Kang et al. 2017) and Lobosa^27^ with the full dataset (113,910 sites). Two loosely constrained topologies Tevosa ([Tubulinea + Evosea] + Discosea) and Lobosa ([Discosea + Tubulinea] + Evosea) were optimized under LG + G4 + F + C60 in IQ-TREE. These optimized trees were compared with our tree (Divosa, ([Discosea + Evosea], Tubulinea) using AU test with 10,000 RELL bootstrap replicates^84^. The hypotheses that had p-AU ≥ 0.05 within the 95% confidence interval could not be rejected.

## Supplementary Information

Supplementary Figure S1.

Supplementary Figure S2.

Supplementary Figure S3.

Supplementary Figure S4.

Supplementary Legends.

Supplementary Information.

Supplementary Table S1.

Supplementary Table S2.

## Data Availability

The datasets analyzed during the current study are available in the NCBI-SRA database. Accession numbers are provided in Table S1. Single gene alignments and trees used in the concatenated analysis are provided in Supplementary file 1.
