# Supplementary figures and images for: New insights on the evolutionary relationships between the major lineages of Amoebozoa
Source: Sci Rep. 2022 Jul 1;12:11173. doi: 10.1038/s41598-022-15372-7 (PMC9249873; doi:10.1038/s41598-022-15372-7)

OBZOA

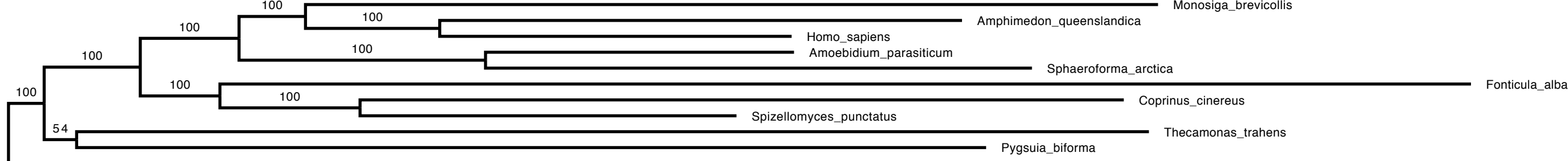

EVOSEA

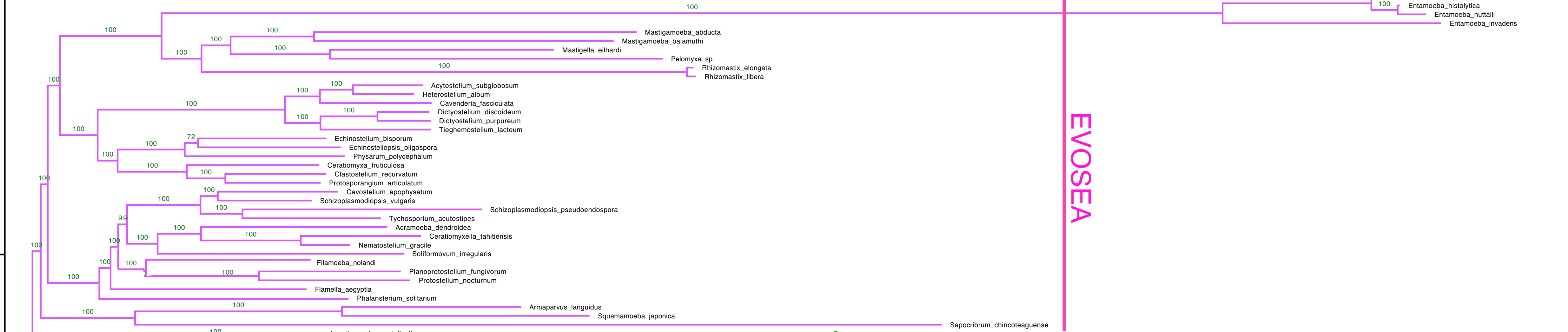

DISCOSEA

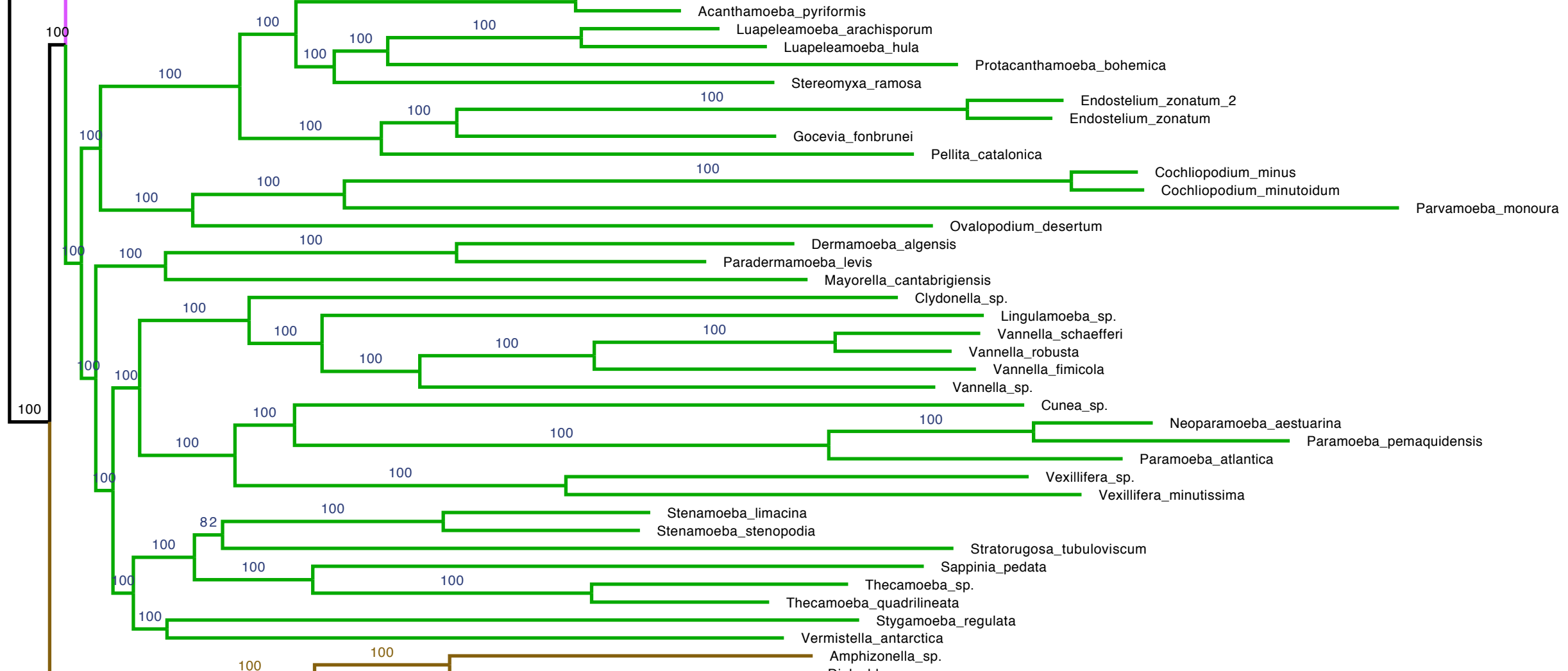

TUBULINEA

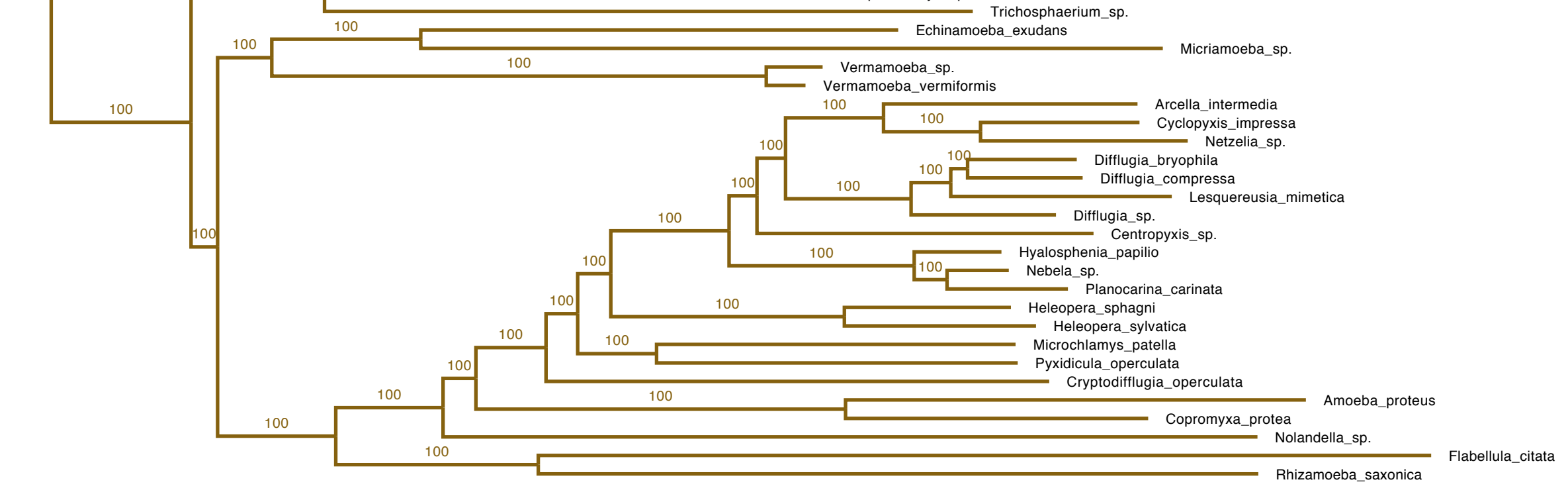

Supplement: Supplementary file 1 — Supplementary Figure S1. [file 41598_2022_15372_MOESM1_ESM.pdf]

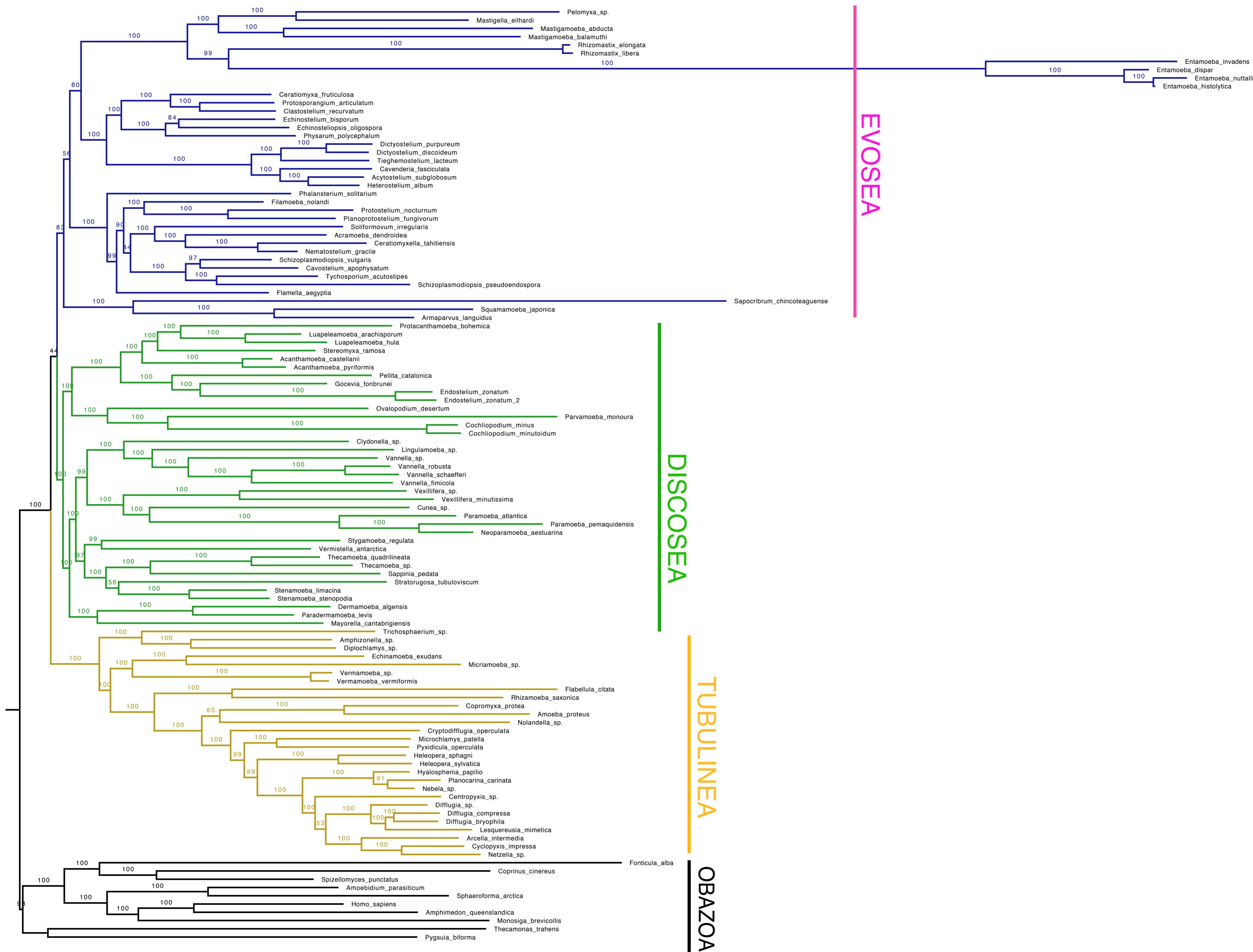

EVOSEA

DISCOSEA

TUBULINEA

OBAZOA

Supplement: Supplementary file 2 — Supplementary Figure S2. [file 41598_2022_15372_MOESM2_ESM.pdf]

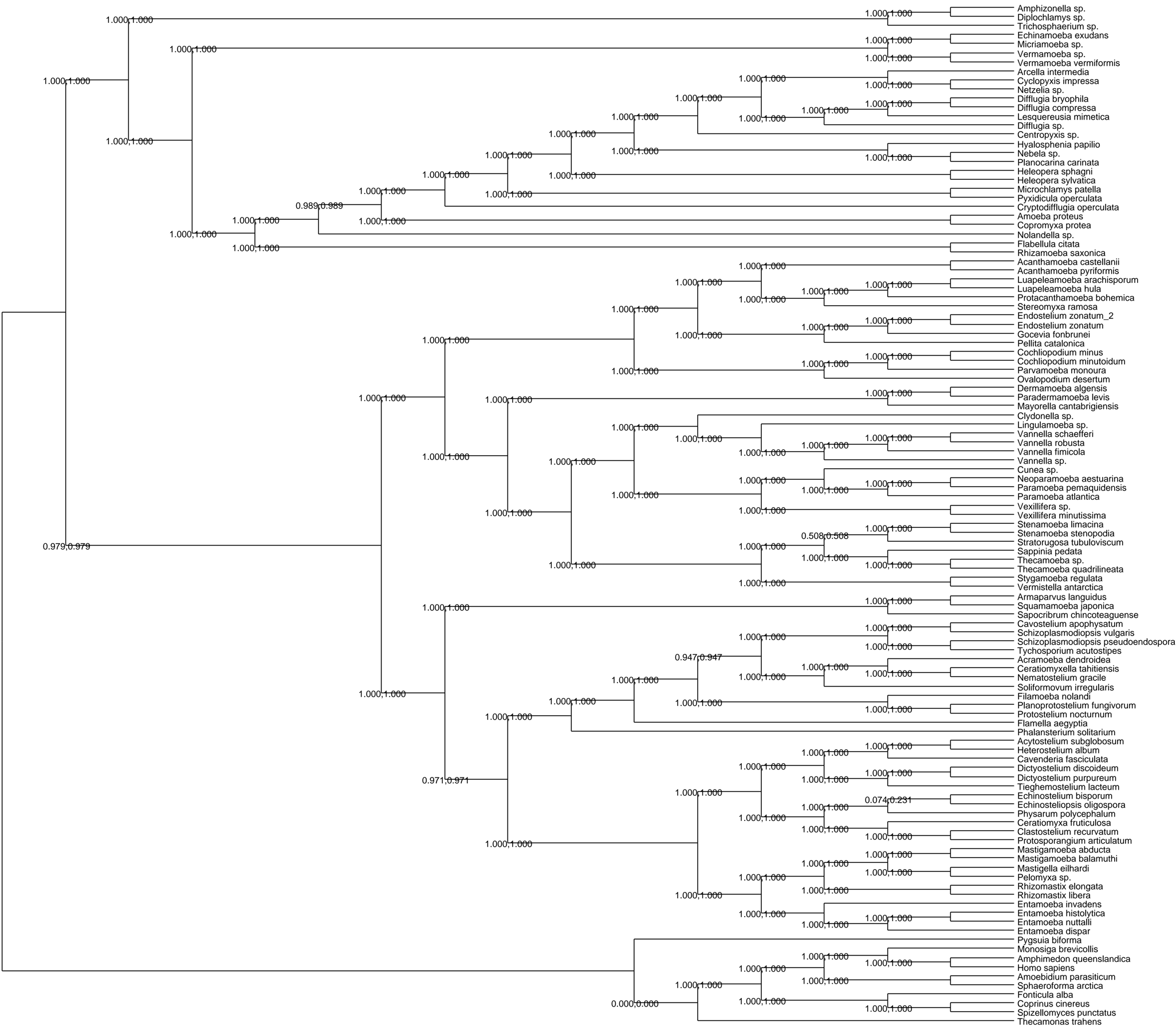

Supplement: Supplementary file 4 — Supplementary Figure S4. [file 41598_2022_15372_MOESM4_ESM.pdf]
