# Supplementary material for: New insights on the evolutionary relationships between the major lineages of Amoebozoa
Source: Sci Rep. 2022 Jul 1;12:11173. doi: 10.1038/s41598-022-15372-7 (PMC9249873; doi:10.1038/s41598-022-15372-7)
Supplement: Supplementary file 5 — Supplementary Legends. [file 41598_2022_15372_MOESM5_ESM.docx]

**Supplementary Figure caption**

**Figure S1**. Genome wide phylogeny of the Amoebozoa inferred using Maximum likelihood (ML) in IQ-TREE with LG+G4+C60+F model of evolution. The data matrix used to infer this tree consisted of 93,820 sites amino acid sites with three fast categories of sites (13%) removed from the full dataset. The data matrix consists of 824 genes and 113 taxa including 10 outgroup taxa. The topology was estimated under LG+G4+C60+F+PMSF [Y1] model using a guide tree from a topology estimated using full dataset shown in Figure 1. Clade supports at nodes are ML IQ-TREE 1000 ultrafast bootstrap values obtained using the same model. All branches are drawn to scale.

**Figure S2**. Maximum Likelihood tree inferred by RAxML with six fast categories of sites removed from the full dataset. The topology was estimated under PROTGAMMALG4X model. Total number of sites included after removing six fast sites categories is 70,543.

**Figure S3**. Internode certainty inferred by QuartetScores for topology in Figure 1. Values at branches are Quadripartition internode certainty (qp-ic); Lowest quartet internode certainty (lp-ic); Extended Quadripartition internode certainty (eqp-ic).

**Figure S4**. Internode certainty inferred using RAxML under PROTGAMMALG4X model for topology in Figure 1. Branch labels showed the internode certainty for a given internode with the most conflicting bipartition (left value) or all conflicting bipartitions (right value). Relative tree certainty including all conflicting bipartitions for this tree is 0.978410.

**Supplementary file 1**. Single gene alignments and corresponding trees of the 824 genes used in the concatenated analysis.
