# Supplementary material for: New insights on the evolutionary relationships between the major lineages of Amoebozoa
Source: Sci Rep. 2022 Jul 1;12:11173. doi: 10.1038/s41598-022-15372-7 (PMC9249873; doi:10.1038/s41598-022-15372-7)
Supplement: Supplementary file 3 — Supplementary Figure S3. [file 41598_2022_15372_MOESM3_ESM.pdf]

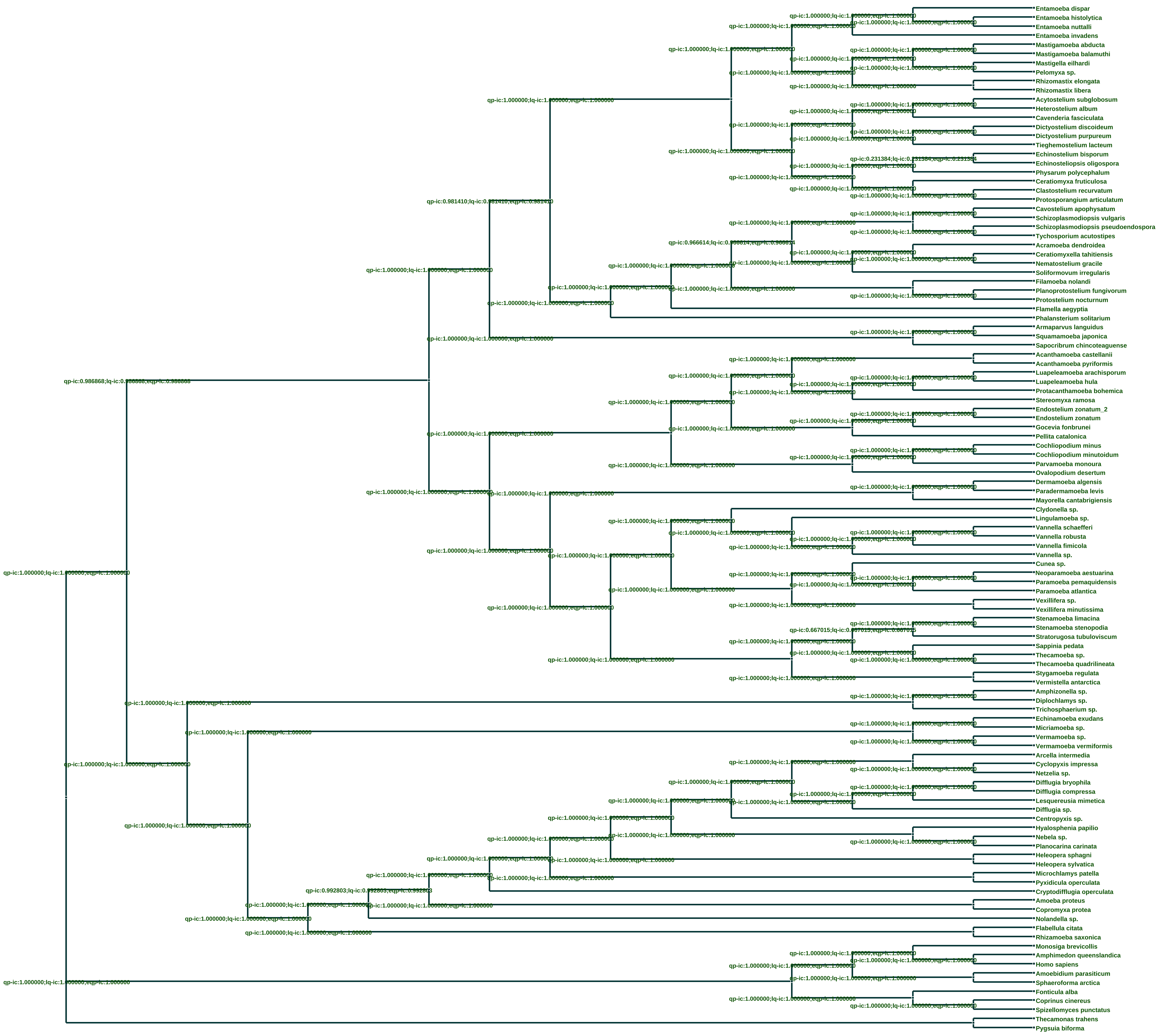
Entamoeba dispar
Entamoeba histolytica
Entamoeba nuttalli
Entamoeba invadens
Mastigamoeba abducta
Mastigamoeba balamuthi
Mastigella eilhardi
Pelomyxa sp.
Rhizomastix elongata
Rhizomastix libera
Acytostelium subglobosum
Heterostelium album
Cavenderia fasciculata
Dictyostelium discoideum
Dictyostelium purpureum
Tieghemostelium lacteum
Echinostelium bisporum
Echinosteliopsis oligospora
Physarum polycephalum
Ceratiomyxa fruticulosa
Clastostelium recurvatum
Protosporangium articulatum
Cavostelium apophysatum
Schizoplasmodiopsis vulgaris
Schizoplasmodiopsis pseudoendospora
Tychosporium acutostipes
Acramoeba dendroidea
Ceratiomyxella tahitiensis
Nematostelium gracile
Soliformovum irregularis
Filamoeba nolandi
Planoprotostelium fungivorum
Protostelium nocturnum
Flamella aegyptia
Phalansterium solitarium
Armaparvus languidus
Squamamoeba japonica
Sapocribrum chincoteaguense
Acanthamoeba castellanii
Acanthamoeba pyriformis
Luapeleamoeba arachisporum
Luapeleamoeba hula
Protacanthamoeba bohemica
Stereomyxa ramosa
Endostelium zonatum_2
Endostelium zonatum
Gocevia fonbrunei
Pellita catalonica
Cochliopodium minus
Cochliopodium minutoidum
Parvamoeba monoura
Ovalopodium desertum
Dermamoeba algensis
Paradermamoeba levis
Mayorella cantabrigiensis
Clydonella sp.
Lingulamoeba sp.
Vannella schaefferi
Vannella robusta
Vannella fimicola
Vannella sp.
Cunea sp.
Neoparamoeba aestuarina
Paramoeba pemaquidensis
Paramoeba atlantica
Vexillifera sp.
Vexillifera minutissima
Stenamoeba limacina
Stenamoeba stenopodia
Stratorugosa tubuloviscum
Sappinia pedata
Thecamoeba sp.
Thecamoeba quadrilineata
Stygamoeba regulata
Vermistella antarctica
Amphizonella sp.
Diplochlamys sp.
Trichosphaerium sp.
Echinamoeba exudans
Micriamoeba sp.
Vermamoeba sp.
Vermamoeba vermiformis
Arcella intermedia
Cyclopyxis impressa
Netzelia sp.
Difflugia bryophila
Difflugia compressa
Lesquereusia mimetica
Difflugia sp.
Centropyxis sp.
Hyalosphenia papilio
Nebela sp.
Planocarina carinata
Heleopera sphagni
Heleopera sylvatica
Microchlamys patella
Pyxidicula operculata
Cryptodifflugia operculata
Amoeba proteus
Copromyxa protea
Nolandella sp.
Flabellula citata
Rhizamoeba saxonica
Monosiga brevicollis
Amphimedon queenslandica
Homo sapiens
Amoebidium parasiticum
Sphaeroforma arctica
Fonticula alba
Coprinus cinereus
Spizellomyces punctatus
Thecamonas trahens
Pygsuia biforma
